# Physical activity and sedentary behavior in preschoolers: a longitudinal assessment of trajectories and determinants

**DOI:** 10.1186/s12966-018-0670-8

**Published:** 2018-04-04

**Authors:** Einat A. Schmutz, Sarah R. Haile, Claudia S. Leeger-Aschmann, Tanja H. Kakebeeke, Annina E. Zysset, Nadine Messerli-Bürgy, Kerstin Stülb, Amar Arhab, Andrea H. Meyer, Simone Munsch, Jardena J. Puder, Oskar G. Jenni, Susi Kriemler

**Affiliations:** 1 Epidemiology, Biostatistics and Prevention Institute, University of Zurich, Hirschengraben 84, 8001 Zurich, Switzerland; 2 Child Development Center, University Children’s Hospital Zurich, Steinwiesstrasse 75, 8032 Zurich, Switzerland; 3 Department of Clinical Psychology and Psychotherapy, University of Fribourg, Clinical Psychology and Psychotherapy, Rue P.A. de Faucigny 2, 1700 Fribourg, Switzerland; 4 Endocrinology, Diabetes & Metabolism Service, Centre Hospitalier Universitaire Vaudois (CHUV), Avenue Pierre Decker 2, 1011 Lausanne, Switzerland; 5 Department of Psychology, University of Basel, Missionsstrasse 62A, 4055 Basel, Switzerland; 6 Division of Pediatric Endocrinology, Diabetology and Obesity, Centre Hospitalier Universitaire Vaudois (CHUV), Rue du Bugnon 46, 1011 Lausanne, Switzerland; 7 Children’s Research Center, University Children’s Hospital Zurich, Steinwiesstrasse 75, 8032 Zurich, Switzerland

**Keywords:** Children, Preschool, Physical activity, Sedentary behavior, Determinants, Trajectories, SPLASHY

## Abstract

**Background:**

Despite physical activity (PA) being recognized as a critically important factor for good physical and mental health already early in life and throughout the life course, prospective data on activity behavior during the preschool years remains scarce. This study examined trajectories and determinants of levels and change in total PA (TPA), moderate-to-vigorous PA (MVPA) and sedentary behavior (SB) in a representative sample of Swiss preschoolers.

**Methods:**

Data were drawn from the Swiss Preschoolers’ Health Study (SPLASHY), a multi-site prospective cohort study including 555 children (53% boys) aged 2-to-6 years at baseline. A follow-up was conducted after 12 months. Activity behavior was measured using accelerometers. Information on 35 potential determinants from different socio-ecological domains was either directly measured or parent-reported. Trajectories of TPA, MVPA and SB over time were described for boys and girls. Linear mixed models were used to investigate factors that predicted levels and change in TPA, MVPA and SB.

**Results:**

All children were sufficiently physically active according to published recommendations for preschoolers. Trajectory profiles revealed a marked increase in TPA and MVPA in boys and girls whereas SB remained fairly stable over time. Mixed modeling demonstrated that variables most relevant to determining PA levels were sex, age and activity temperament (all positively associated). Together with gross motor skills, birth weight, family structure (only for TPA) and season (only for MVPA), these factors accounted for 26 and 32% of total variance explained in TPA and MVPA, respectively. Activity temperament emerged as the strongest determinant of SB (negative association) and explained with sex, season and family structure 20% of total variance in SB. The presence of older siblings was the only factor that predicted change in PA over time.

**Conclusions:**

In this healthy physically active cohort of preschoolers, non-modifiable individual-level factors had the greatest influence on PA. The limited success of this and previous studies to identify modifiable determinants and the finding that most preschoolers were sufficiently active suggest that future attempts should provide insights into how preschoolers’ activity levels can be maintained and fostered to prevent subsequent harmful declines attributable, amongst others, to educational transitions. Thus, good-quality longitudinal studies are needed.

**Trial registration:**

Current Controlled Trials ISRCTN41045021 (date of registration: 21.03.14).

**Electronic supplementary material:**

The online version of this article (10.1186/s12966-018-0670-8) contains supplementary material, which is available to authorized users.

## Background

Increased physical activity (PA) in the preschool age has been shown to be positively associated not only with decreased adiposity, but also with improved measures of psychosocial health, motor skill development, and cardio-metabolic health indicators [1]. Thus, it is recommended that preschoolers engage in PA every day for at least 3 h and minimize the amount of time spent being sedentary [2–4]. Whether young children are sufficiently physically active and how PA changes over time during the preschool years is unclear; while a number of studies have reported engagement in sufficient levels of PA [5–7], others have found that preschool children do not meet the guidelines [8, 9]. Moreover, two studies in children aged 3 to 4 years at baseline showed an increase in PA over 1 to 2 years [10, 11], whereas others found substantial declines [12].

Healthy habits and behaviors, such as PA and sedentary behavior (SB), are typically established during early childhood. Evidence suggests that these behaviors track into later life [13]. To support establishing healthy levels of PA and SB in childhood, the early identification of factors associated with these behaviors is an important research focus [1, 14]. Since PA is a complex behavior with different levels of influence, the socio-ecological model [15] has been widely used to structure the study of potential correlates and determinants. Applying a social-ecological perspective, variables from different domains (demographic and biological; psychological, cognitive and emotional; behavioral; social and cultural; and environmental) can be explored. Numerous studies have examined potential influences in order to understand children’s PA behavior and develop more targeted interventions. However, previous interventional studies aiming at promoting PA in children have had limited efficacy [16, 17]. A possible explanation why past attempts have largely proven unsuccessful involves the meaningful interpretation of evidence, which has mainly relied on cross-sectional data [16]. Such analyses take measurements of the outcome variable at only one time point into account. Where outcome measurements from additional time points are available, data are considered to be longitudinal and corresponding mixed model regression analysis can be applied. These models use all available measurements and take multiple measurements from each subject into account, resulting in more reliable results than using simple cross-sectional data. Whereas a large number of observational studies examined the correlates of PA in the early years, few have provided longitudinal data. Thus, to advance the field and establish a robust evidence base that can inform the design of effective behavior change interventions, research has called for studies that employ a prospective design to explore the whole spectrum of the social-ecological model within one study [16, 18].

Results from the few previous longitudinal studies on determinants of young children’s PA levels have been inconsistent. A recent systematic review on determinants during the early years (age 0-6 years) found only sex and time spent playing to be positively associated with TPA while no consistent determinant was identified for MVPA [16]. Regarding SB, there has not been enough evidence to draw conclusions [19, 20]. It is important to recognize that determinants of activity levels may differ from determinants of rates of change in these levels. For a full understanding of the complexities with regard to participation in, barriers to, and preferences for PA in this age group, predictors of activity levels (subsequently called *determinants*) as well as those of changes in these levels (subsequently called *determinants of change*) is required. A recent publication synthesizing quantitative literature from longitudinal studies in preschool-aged children concluded that of 44 studied determinants of change only parental monitoring of their child’s physical activity was consistently associated with change in physical activity [21]. Childcare provider training emerged as a determinant of change in MVPA.

Building upon previous work, the aim of the current study was threefold: (1) to describe trajectories of TPA, MVPA and SB over time in boys and girls, (2) to examine associations between potential determinants and TPA, MVPA and SB, and (3) to investigate associations between potential determinants of change and TPA, MVPA and SB.

## Methods

### Study population and data collection

Data were drawn from the Swiss Preschoolers’ Health Study (SPLASHY), a multi-site prospective cohort study including 555 children aged 2 to 6 years from 84 childcare centers located in five cantons of Switzerland (covering 50% of the Swiss population in 2013). A detailed description of the study design has been published elsewhere [22]. Data collection was conducted in the childcare centers in 2014 and 1 year later by the same study team in parallel at all study sites. While children recruited in 2014 (*n* = 476) could participate in a follow-up assessment 1 year later, those recruited in 2015 (*n* = 79) had a baseline assessment only. Baseline (T0) and follow-up (T1) data are used in the current study. Ethical approval is in accordance with the Declaration of Helsinki and has been obtained from all local ethical committees, with the Ethical Committee of the Canton of Vaud (No 338/13) being the main approving authority. Children and parents provided informed consent.

### Outcome variables: TPA, MVPA and SB

PA and SB were objectively monitored for seven consecutive days using a hip-worn accelerometer measuring tri-axial acceleration (wGT3X-BT, ActiGraph, Pensacola, FL, USA). At both assessment time points, participants were instructed to wear the monitor 24 h/day except during water-based activities. PA data was sampled at a frequency of 30 Hz, downloaded in three-second epochs and aggregated to 15-s epochs. Non-wear periods, defined as ≥20 min of consecutive zero counts on all axes [23], and nighttime hours (9 pm to 7 am) were excluded. A minimum of 3 days, including one weekend day, with at least 10 h of recording per day were required for inclusion in analysis. Accelerometry data were expressed as TPA (accelerometry counts per min [cpm], averaged over the recording time) and as time spent at different activity intensities (min/day). Besides comparability considerations, accelerometer cut-points were chosen based on recent findings [24] showing that best classification accuracy has been achieved for the validated pediatric cut-points of Pate RR, Almeida MJ, McIver KL, Pfeiffer KA and Dowda M [25] for MVPA (≥420 counts per 15 s) and Evenson KR, Catellier DJ, Gill K, Ondrak KS and McMurray RG [26] for SB (≤25 counts per 15 s).

### Potential determinants

In this work, the term determinant is used to describe a factor whose variation is followed systematically by variations in PA [27]. Previous research and the socio-ecological model guided the selection of potential determinants, which were classified into five domains [23]: (i) biological and demographic; (ii) psychological, cognitive and emotional; (iii) behavioral; (iv) social and cultural; and (v) environmental. A detailed description of all potential determinants is provided in Additional file 1.

### Statistical analysis

Descriptive statistics are presented as mean (standard deviation [SD]) for continuous variables and percentages for categorical variables, unless stated otherwise. To describe trajectories in PA and SB on the population level (aim 1), we present observed mean levels of TPA, MVPA and SB for different age categories ranging from two-and-a-half to six-and-a-half years for boys and girls separately. Differences between age groups were tested for using linear mixed models with categorical age as a single fixed effect and random intercepts for both child and childcare center. To examine associations between potential determinants and TPA, MVPA and SB over all time points (aim 2), linear mixed models for the outcome (at either time point) were used. Potential determinants, time point of assessment and accelerometer wear time were entered into the model simultaneously as fixed effects. Random intercepts were included for each childcare center and each subject (nested within childcare center). Factors for which there was at least some indication for an association with the outcome (i.e., *p* ≤ 0.10) were subsequently included in the final model. Associations between potential determinants (assessed at T0) of changes in TPA, MVPA and SB from baseline to follow-up (aim 3) were examined by linear mixed modeling including a random intercept for childcare center. A change score could be calculated for *n* = 454. One alternative model would have been ANCOVA (PA at T1 as a function of PA at T0 and other covariates) but we wanted to model the effects of potential determinants on the change in PA directly. To avoid regression to the mean, we regressed the change on the average of the baseline and follow-up PA instead of adjusting for baseline values [28]. All analysis steps for aim 1, 2 and 3 were defined a priori and not changed or added to after examining the results.

Collinearity diagnostics indicated that no significant multicollinearities were present. *P*-values obtained in the final models were used to quantify the explanatory power of potential determinants, i.e. the smaller the *p*-value, the stronger the evidence for an association with the outcome. R^2^ was calculated for the final models to capture the amount of variance explained; Marginal R^2^ (variance explained by fixed effects) and conditional R^2^ (variance explained by fixed and random effects) were estimated as described by Nakagawa S and Schielzeth H [29]. To check the form of the association between sex and age and a potential interaction, we considered two models: (a) age and sex as main effects and (b) age and sex as main effects plus their interaction. Since these analyses led to comparable results, only the main effects model (a) is presented here.

Missing data in both outcome and covariates were imputed using the MICE (Multiple Imputation Chained Equations) procedure [30]. While a complete case analysis may be straightforward to implement, it relies on stronger missing data assumptions than multiple imputation (MI) and can result in biased and less powerful estimates [31, 32]. The imputation model contained all variables included in the analysis model and the form of the variables was the same in both models [33]. There were no differences in key baseline characteristics (BMI, SES, siblings, parental BMI and family structure) between individuals with complete and incomplete data. Furthermore, there was no evidence of statistical heterogeneity between observed and imputed values and results from complete case analysis did not substantially differ from those based on MI (40 imputed datasets). A total of 158 measurement time points of children who did not have at least one valid PA measurement were excluded from analysis after imputation, resulting in a final sample of 498 children providing 952 observation time points (see Additional file 2 for a flow chart). Results presented in this work are based on imputed data, unless stated otherwise. No adjustments were made for multiple testing [34]. All analyses were performed using R, version 3.2.3 (R Foundation for Statistical Computing, Vienna, Austria).

## Results

Data for the current analysis are from the baseline (T0) and follow-up (T1) assessment of the SPLASHY study. Descriptive statistics of included participants (*n* = 498) are shown in Table 1. Mean age was 3.9 (0.7) and 4.9 (0.7) years at T0 and T1, respectively. All children at both time points fulfilled the recommendations of engaging in at least 180 min of PA per day. Children who participated at T1 and those lost to follow-up did not differ by age or sex.

**Table 1:** Participant characteristics and potential determinants of young children’s objectively measured physical activity and sedentary behavior (*n* = 498)

		Mean (SD) or percentages	
	Use in analysis	T0	T1
Demographic and biological variables			
Sex	% boys	53.8	51.9
Age	years	3.9 (0.7)	4.9 (0.7)
Birth weight	grams (per 100 g)	3290 (570)	3280 (530)
Chronic health condition	% with chronic health condition	7.5	7.1
BMI^a^	% overweight or obese	24.1	18.5
Gross motor skills^a^	Composite z-score	0.0 (1.0)	0.1 (1.1)
Siblings	% having older siblings	43.2	44.5
Parental BMI	% at least one overweight or obese parent	52.2	52.5
SES	Range 16–90; increases with higher SES	63.1 (15.5)	62.7 (15.6)
Family structure	% single parent households	10.4	9.8
Psychological, cognitive and emotional variables			
Self-regulation^a^	Range 0-30; increases with better self-regulation	19.2 (9.5)	23.3 (8.0)
Psychological difficulties	Range 0-40; increases with more difficulties	8.8 (4.3)	8.5 (4.7)
Emotionality temperament	Range 1-5; increases with more pronounced trait	2.8 (0.7)	2.8 (0.7)
Activity temperament	Range 1-5; increases with more pronounced trait	3.8 (0.7)	3.7 (0.7)
Shyness temperament	Range 1-5; increases with more pronounced trait	2.4 (0.7)	2.4 (0.7)
Parenting stress	Range 5-90; increases with more parenting stress	37.5 (7.5)	37.6 (7.6)
Cognitive performance^a^	Composite z-score	0.0 (1.0)	0.1 (0.9)
Behavioral variables			
Sleep duration	Hours	10.8 (0.6)	10.8 (0.5)
Play frequency	% more than once/week	91.2	93.1
Social and cultural variables			
Parental sedentary behavior	Hours	2.8 [2.0, 4.5]^b^	3.0 [2.0, 5.0]^b^
Parental sports club membership	% at least one parent is member	32.0	32.8
Parental physical activity	% at least one parent is active	63.4	61.6
Parental Involvement in child physical activity	% at least one parent is involved	65.0	52.3
Mode of transport to childcare	% active	40.7	48.1
Parental tobacco use	% at least one parent smokes	24.1	24.4
Parental alcohol consumption	% at least one parent consumes large amounts	5.5	4.5
Environmental variables			
Time outdoors	Hours	2.0 [1.5, 3.0]^b^	2.0 [1.5, 3.0]^b^
Fixed toys	Range 0-7	1.6 (1.5)	1.6 (1.6)
Portable toys	Range 0-8	4.5 (1.5)	4.3 (1.5)
Days at childcare	Range 0-5	2.8 (1.2)	2.5 (1.3)
Living area per person	m^2^	30.8 (9.1)	30.8 (8.9)
Neighborhood safety	Range 0-44; increases with increasing concerns	12.6 (7.0)	12.4 (7.2)
Dog	% dog owner	6.9	5.5
Season	% spring and fall months	75.3	77.9
Region	% urban	34.9	39.4

*PA* physical activity, *SB* sedentary behavior, *BMI* body mass index, *SES* socio-economic status

^a^Directly measured (all other information is parent-report)

^b^Median and inter-quartile range presented for skewed distribution

TPA and MVPA increased with increasing age in both boys and girls (Table 2) with aggregated levels being significantly higher at 4, 5 and 6 years compared to those observed at 3 years (aim 1).

**Table 2:** Total physical activity (TPA), moderate-to-vigorous physical activity (MVPA) and sedentary behavior (SB) at each age category

	3 Years^a^	4 Years^a^	5 Years^a^	6 Years^a^
N_total_ (N_girls_, N_boys_)	147 (75, 72)	397 (192, 205)	322 (144, 178)	86 (38, 48)
Number of valid days	5.7 (1.7)	5.6 (1.4)	5.7 (1.6)	5.8 (1.3)
Monitor wear time [hours/day]	12.7 (0.7)	12.8 (0.8)	12.9 (0.8)	13.0 (0.8)
TPA [cpm]				
Boys	595.2 (138.4)	654.9 (160.3)	681.5 (148.5)	731.1 (167.3)
Girls	560.9 (132.6)	604.7 (142.6)	613.5 (142.6)	616.6 (154.7)
All	577.8 (136.2)	631.3 (154.1)	651.6 (149.5)	683.3 (170.7)
MVPA [min/day]				
Boys	86.5 (27.6)	101.2 (28.3)	107.5 (28.2)	118.5 (31.7)
Girls	76.1 (25.3)	85.7 (25.4)	90.3 (26.6)	91.4 (23.0)
All	81.2 (26.9)	93.9 (28.0)	99.9 (28.8)	107.2 (31.2)
SB [min/day]				
Boys	370.7 (47.6)	368.0 (49.2)	366.0 (45.9)	370.3 (50.4)
Girls	377.4 (46.1)	376.1 (51.3)	377.4 (53.5)	395.7 (46.3)
All	374.1 (46.8)	371.8 (50.3)	371.0 (49.6)	380.9 (50.0)

Aggregated data on the population level. Data are presented as mean (SD) except for N

^a^Age categories are rounded to +/− 6 months, e.g. 3 years corresponds to 2.5-3.5 years

Boys in the highest age category (6 years) accumulated 23% more TPA and 37% more MVPA and spent around the same amount of time sedentary as the youngest children (3 years). Likewise, girls in the highest age category accumulated 10% more TPA and 20% more MVPA and spent 5% less time sedentary compared to the youngest. The relative increase of mean TPA levels was significant in both boys and girls from 3 to 4 years. From four to five only boys showed a further significant increase and from five to six the change was non-significant in both sexes. For MVPA the increase was significant in both boys and girls from 3 to 4 and 4 to 5 years, without any further significant increase occurring between 5 and 6 years. Time spent sedentary (around 6 h per day) remained reasonably stable over time with an average change of less than 2% comparing the oldest to the youngest age category.

**Fig. 1:**
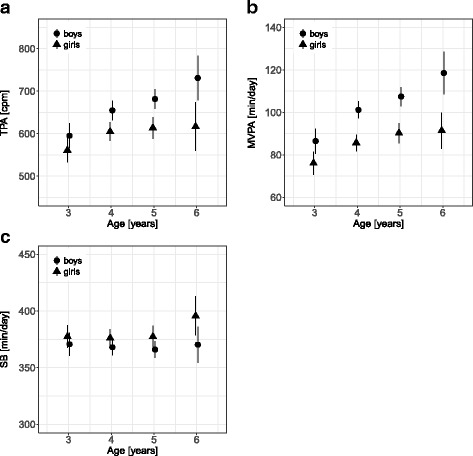
Physical activity trajectories by age. Observed mean **a** total physical activity (TPA), **b** moderate-to-vigorous physical activity (MVPA), and **c** sedentary behavior (SB) by age with 95% confidence intervals (vertical lines), separately for boys (circle) and girls (triangle)

To illustrate PA progression over time, observed values were plotted by age (Fig. 1). Trajectory profiles indicated that the difference between boys and girls gradually increased up to a difference of 115 cpm (16%) in TPA and 27 min/day (24%) in MVPA at age six.

Table 3 presents multivariate associations between potential determinants of TPA, MVPA and SB (aim 2).

**Table 3:** Full models: associations of potential determinants with total physical activity (TPA), moderate-to-vigorous physical activity (MVPA) and sedentary behavior (SB)^a^

	TPA [cpm]			MVPA [min/day]			SB [min/day]		
	β	95% CI	p-value	β	95% CI	p-value	β	95% CI	p-value
Demographic and biological variables									
Sex	46.7	(24.4, 68.9)	≤0.001	14.4	(10.1 18.7)	≤0.001	−5.1	(−13.1, 2.8)	0.208
Age	32.5	(12.4, 52.6)	0.002	8.4	(4.6, 12.1)	≤0.001	0.4	(−6.5, 7.3)	0.906
Birth weight	2.2	(0.2, 4.2)	0.035	0.4	(0.1, 0.8)	0.030	−0.6	(−1.4, 0.1)	0.077
Chronic health condition	4.1	(−38.4, 46.5)	0.850	0.2	(−7.5, 7.9)	0.959	−2.2	(−17.6, 13.2)	0.779
BMI	9.8	(−16.0, 35.6)	0.457	2.7	(−2.0, 7.5)	0.261	−5.1	(−14.6, 4.3)	0.286
Gross motor skills	13.4	(2.1, 24.7)	0.021	2.9	(0.9, 4.8)	0.005	−1.3	(−5.2, 2.6)	0.515
Siblings	4.8	(−17.9, 27.6)	0.676	1.8	(−2.7, 6.3)	0.431	−1.6	(−9.5, 6.3)	0.694
Parental BMI	−0.9	(−33.2, 24.4)	0.941	0.8	(−3.4, 5.1)	0.706	−2.1	(−10.2, 5.9)	0.604
SES	0.1	(−0.8, 1)	0.849	0.0	(−0.2, 0.1)	0.837	−0.2	(−0.5, 0.2)	0.332
Family structure	50.8	(6.1, 95.6)	0.027	6.2	(−2.1, 14.5)	0.142	−17.3	(−33.7, −0.9)	0.039
Psychological, cognitive and emotional variables									
Self-regulation	−0.2	(−1.6, 1.1)	0.724	0.0	(−0.3, 0.2)	0.896	0.2	(−0.2, 0.7)	0.302
Psychological difficulties	1.4	(−1.7, 4.4)	0.373	0.4	(−0.2, 0.9)	0.210	−0.2	(−1.3, 0.9)	0.712
Emotionality temperament	−12.1	(−30.2, 6.1)	0.191	−2.2	(−5.6, 1.2)	0.200	4.5	(−1.6, 10.5)	0.148
Activity temperament	30.6	(12.7, 48.5)	≤0.001	5.4	(1.9, 8.9)	0.002	−11.8	(−18.3, −5.3)	< 0.001
Shyness temperament	−10.4	(−26.9, 6.2)	0.218	−1.5	(−4.6, 1.5)	0.317	1.4	(−4.2, 7.1)	0.623
Parenting stress	2.0	(0.4, 3.6)	0.015	0.2	(−0.1, 0.5)	0.126	−0.4	(−1.0, 0.1)	0.130
Cognitive performance	−0.6	(−12.3, 11.1)	0.916	0.7	(−1.4, 2.9)	0.501	0.8	(−3.4, 5.0)	0.714
Behavioral variables									
Sleep duration	−5.3	(−24.6, 13.9)	0.585	−1.1	(−4.6, 2.4)	0.614	−1.7	(−8.2, 4.8)	0.599
Play frequency	−10.3	(−50.2, 29.5)	0.609	−1.7	(−8.6, 5.2)	0.911	5.0	(−11.2, 21.3)	0.540
Social and cultural variables									
Parental sedentary behavior	−0.1	(−3.8, 3.7)	0.976	0.1	(−0.6, 0.8)	0.791	0.2	(−1.1, 1.5)	0.800
Parental sports club membership	10.2	(−14.1, 34.4)	0.410	0.4	(−4.0, 4.8)	0.854	−4.5	(−13.0, 4.1)	0.307
Parental physical activity	−8.7	(−31.9, 14.5)	0.459	−1.3	(−5.6, 3.0)	0.547	−0.7	(−9.2, 7.7)	0.866
Parental Involvement in child PA	8.0	(−14.1, 30.1)	0.477	1.3	(−2.8, 5.4)	0.535	−2.7	(−10.6, 5.2)	0.496
Transport to childcare	6.2	(−15.6, 28.0)	0.576	2.0	(−2.0, 6.1)	0.326	−4.7	(−12.4, 3.0)	0.233
Parental tobacco use	−5.1	(−32.1, 21.9)	0.710	−0.3	(−5.3, 4.7)	0.897	3.3	(−6.1, 12.8)	0.487
Parental alcohol consumption	7.3	(−40.9, 55.4)	0.765	2.6	(−6.6, 11.8)	0.580	−8.0	(−26.9, 10.9)	0.402
Environmental variables									
Time outdoors	4.6	(−2.6, 11.8)	0.205	0.2	(−1.1, 1.5)	0.807	−1.4	(−3.9, 1.0)	0.247
Fixed toys	3.8	(−3.7, 11.3)	0.318	0.1	(−1.3, 1.5)	0.907	−0.5	(−3.2, 2.2)	0.713
Portable toys	4.8	(−3.5, 13.2)	0.253	0.7	(−0.7, 2.2)	0.339	−2.6	(−5.4, 0.2)	0.067
Days at childcare	1.8	(−9.6, 13.2)	0.751	−0.1	(−2.1, 1.9)	0.924	0.7	(−3.3, 4.8)	0.726
Living area per person	0.6	(−0.8, 2.0)	0.415	0.1	(−0.2, 0.4)	0.432	0.0	(−0.5, 0.5)	0.911
Neighborhood safety	−0.9	(−2.8, 0.9)	0.330	−0.1	(−0.5, 0.2)	0.481	0.1	(−0.5, 0.8)	0.730
Dog	0.5	(−42.5, 43.6)	0.980	−0.8	(−8.7, 7.2)	0.853	−1.3	(−16.4, 13.8)	0.864
Season	4.1	(−22.2, 30.5)	0.758	4.1	(−0.9, 9.0)	0.106	9.1	(−0.8, 18.9)	0.071
Region	1.7	(−23.4, 26.8)	0.892	0.5	(−4.3, 5.2)	0.852	−1.3	(−10.4, 7.8)	0.782

*PA* physical activity, *β* β-coefficient, *CI* confidence interval

^a^Multivariable linear mixed models including a random intercept for each subject and for each childcare center (*n* = 498)

All seven variables associated with TPA in the full multilevel analysis (*p* ≤ 0.1) were identified as determinants in the final model (all *p* ≤ 0.041; Table 4). Boys were more active than girls and TPA was positively associated with age, gross motor skills and activity temperament. Children from single-parent families were more active than those living with two parents. For MVPA, five of a total of seven variables associated with the outcome in the full model were identified as determinants in the final model (all *p* ≤ 0.027). Similar to TPA, boys spent more time moderately-to-vigorously active, MVPA increased with age, gross motor skills and activity temperament. Birth weight and parenting stress were also identified as determinants of both TPA and MVPA (positive association).

**Table 4:** Final models: associations of determinants with total physical activity (TPA), moderate-to-vigorous physical activity (MVPA) and sedentary behavior (SB)^a^

	TPA [cpm]			MVPA [min/day]			SB [min/day]		
	β	95% CI	p-value	β	95% CI	p-value	β	95% CI	p-value
Demographic and biological variables									
Sex	49.8	(28.5, 71.2)	≤0.001	14.8	(10.7, 19.0)	≤0.001	−7.1	(−14.4, 0.3)	0.062
Age	31.6	(17.6, 45.6)	≤0.001	8.9	(6.0, 11.8)	≤0.001	–	–	–
Birth weight	2.3	(0.4, 4.2)	0.018	0.4	(0.1, 0.8)	0.027	–	–	–
Gross motor skills	10.7	(0.1, 21.3)	0.048	2.2	(0.4, 4.2)	0.023	–	–	–
Family structure	59.3	(11.7, 106.8)	0.016	–	–	–	−20.1	(−46.0, 5.7)	0.123
Psychological, cognitive and emotional variables									
Activity temperament	39.2	(23.2, 55.2)	≤0.001	6.4	(3.1, 9.6)	≤0.001	−12.8	(−19.1, −6.5)	≤0.001
Parenting stress	1.8	(0.3, 3.2)	0.017	0.2	(−0.1, 0.5)	0.123	–	–	–
Environmental variables									
Season	–	–	–	2.6	(−2.8, 7.9)	0.344	11.9	(1.2, 22.6)	0.029

*β* β-coefficient, *CI* confidence interval

^a^Multivariable linear mixed models including a random intercept for each subject and for each childcare center showing all variables with *P* ≤ 0.1 of the respective full model (*n* = 498)

With SB as the outcome, results of the final model indicated that for two of four variables taken from the full model there was evidence for an association (all *p* ≤ 0.029): activity temperament was negatively associated with SB and children were shown to be more sedentary in the spring and fall months compared to summer. The proportion of variance explained by the fixed factors (marginal R^2^) in the final models for TPA, MVPA and SB was 16%, 18% and 9%, respectively. Including the random factors (conditional R^2^) resulted in 26%, 32% and 20% of variance explained in TPA, MVPA and SB. The increase in explained variance seen when adding the random factors was mainly attributable to the measurements nested within the child rather than the childcare center.

Although a number of factors were associated with levels of PA, as noted above, only one variable was shown to be associated with change in PA over time (aim 3; Table 5). Specifically, having an older sibling at baseline was associated with a difference in change of about 50 cpm for TPA and 10 min/day for MVPA. None of the variables were associated with change in SB.

**Table 5:** Change models: associations of potential determinants with changes in total physical activity (TPA), moderate-to-vigorous physical activity (MVPA) and sedentary behavior (SB)^a^

	TPA [cpm]			MVPA [min/day]			SB [min/day]		
	β	95% CI	p-value	β	95% CI	p-value	β	95% CI	p-value
Demographic and biological variables									
Sex	22.2	(−13.2, 57.7)	0.218	4.4	(−2.3, 11.1)	0.195	−8.0	(−21.2, 5.2)	0.238
Age	−28.4	(−66.2, 9.3)	0.139	−5.6	(−12.3, 1.2)	0.108	−3.9	(−17.5 9.7)	0.571
Birth weight	0.4	(−3.1, 3.9)	0.817	0.0	(−0.6, 0.6)	0.996	−0.3	(−1.4, 0.9)	0.660
Chronic health condition	2.9	(−67.4,73.2)	0.936	2.2	(−10.2, 14.7)	0.726	−4.6	(−30.7, 21.4)	0.726
BMI	−26.5	(−68.9, 15.9)	0.220	−3.9	(−11.4, 3.5)	0.303	4.1	(−12.2, 20.3)	0.622
Gross motor skills	−16.8	(−37.6, 3.9)	0.111	−3.0	(−6.6, 0.6)	0.111	5.3	(−1.7, 12.3)	0.142
Siblings	50.5	(12.4, 88.6)	0.010	10.1	(3.1, 17.0)	0.004	−8.2	(−23.3, 6.9)	0.287
Parental BMI	−11.0	(−48.6, 26.6)	0.566	−3.0	(−9.7, 3.7)	0.389	6.2	(−8.2, 20.6)	0.395
SES	0.0	(−1.3, 1.4)	0.969	0.0	(−0.2, 0.2)	0.792	0.0	(−0.5, 0.4)	0.858
Family structure	−7.3	(−79.9, 65.3)	0.842	−1.1	(− 13.6, 11.4)	0.864	17.8	(−6.9, 42.6)	0.158
Psychological, cognitive and emotional variables									
Self-regulation	0.4	(−1.8, 2.6)	0.735	0.1	(−0.3, 0.4)	0.683	0.1	(−0.7, 0.87)	0.833
Psychological difficulties	−4.4	(−9.7, 0.9)	0.105	−0.9	(−1.8, 0.1)	0.076	0.5	(−1.4, 2.5)	0.587
Emotionality temperament	11.1	(−21.2, 43.4)	0.497	2.1	(−3.7, 7.9)	0.480	−3.3	(−15.1, 8.6)	0.588
Activity temperament	−8.0	(−39.1, 23.1)	0.612	−0.1	(−5.8, 5.6)	0.976	6.8	(−4.1, 17.8)	0.221
Shyness temperament	0.5	(−28.2, 29.2)	0.974	0.4	(−4.7, 5.4)	0.880	2.2	(−7.9, 12.2)	0.672
Parenting stress	−0.5	(−3.2, 2.2)	0.719	−0.1	(−0.5, 0.4)	0.742	0.1	(−0.9, 1.1)	0.828
Cognitive performance	−4.7	(−24.1, 14.7)	0.637	−2.2	(−5.6, 1.2)	0.203	−0.7	(−7.6, 6.2)	0.842
Behavioral variables									
Sleep duration	−3.0	(−34.7, 28.7)	0.853	0.1	(−5.5, 5.6)	0.984	4.7	(−5.6, 14.9)	0.375
Play frequency	53.5	(−10.9, 117.8)	0.103	7.2	(−4.2, 18.6)	0.216	−11.9	(−36.4, 12.5)	0.337
Social and cultural variables									
Parental sedentary behavior	−0.9	(−6.9, 5.1)	0.761	− 0.2	(−1.3, 0.8)	0.670	0.5	(−1.7, 2.7)	0.683
Parental sports club membership	3.9	(−41.3, 49.1)	0.866	2.6	(−5.5, 10.7)	0.531	7.3	(−9.0, 23.6)	0.376
Parental physical activity	−6.8	(−47.3, 33.8)	0.743	−0.5	(−7.7, 6.7)	0.898	3.5	(−11.1, 18.2)	0.637
Parental Involvement in child PA	−9.8	(−51.3, 31.7)	0.643	−2.9	(−9.9, 4.2)	0.428	0.3	(−15.3, 15.9)	0.968
Transport to childcare	−0.5	(−39.5, 38.6)	0.981	−0.7	(−7.6, 6.1)	0.836	3.7	(−10.4, 17.8)	0.610
Parental tobacco use	−4.0	(−48.2, 40.2)	0.859	−0.5	(−8.5, 7.5)	0.902	1.7	(−15.8, 19.1)	0.853
Parental alcohol consumption	−12.9	(−98.2, 72.3)	0.764	−3.6	(−18.4, 11.1)	0.627	8.4	(−22.9, 39.6)	0.598
Environmental variables									
Time outdoors	−8.1	(−21.5, 5.2)	0.233	−0.5	(−2.93, 1.9)	0.705	2.4	(−2.5, 7.3)	0.335
Fixed toys	−5.8	(−19.2, 7.5)	0.392	−0.6	(−3.0, 1.7)	0.613	0.6	(−4.4, 5.5)	0.827
Portable toys	3.5	(−11.8, 18.9)	0.651	0.5	(−2.2, 3.3)	0.695	−1.6	(−7.2, 4.0)	0.574
Days at childcare	0.7	(−17.8, 19.1)	0.944	0.2	(−3.1, 3.4)	0.919	−0.4	(−7.0, 6.2)	0.907
Living area per person	0.8	(−1.5, 3.1)	0.519	0.1	(−0.3, 0.4)	0.791	−0.4	(−1.3, 0.4)	0.331
Neighborhood safety	1.2	(−1.6, 4.1)	0.402	0.1	(−0.4, 0.6)	0.691	−0.1	(−1.1, 1.0)	0.921
Dog	15.6	(−55.2, 86.4)	0.664	3.6	(−9.4, 16.6)	0.584	−6.4	(−32.9, 20.2)	0.637
Season	−31.1	(−76.4, 14.3)	0.178	−4.0	(−12.4, 4.5)	0.356	3.7	(−12.6, 20.0)	0.657
Region	1.1	(−43.7, 46.0)	0.961	0.3	(−7.9, 8.6)	0.938	−5.8	(−21.3, 9.7)	0.461

*PA* physical activity, *β* β-coefficient, *CI* confidence interval

^a^Multivariable linear mixed models including a random intercept for each childcare center (*n* = 394)

## Discussion

The current study extends the extensive cross-sectional literature on preschoolers’ activity behavior by examining longitudinal trajectories and associations of PA and SB in a representative preschool population. We found that PA increased over time in this active and healthy cohort with the increase being more pronounced in boys than girls. Non-modifiable individual-level variables had the greatest influence on PA levels; Sex and age were shown to be major determinants of TPA and MVPA and activity temperament was consistently strongly associated with all three outcomes. Gross motor skills were positively associated with both, TPA and MVPA, and family structure was shown to play a role in TPA, that is children of single parents were more active than those living in dual-parent families. The only determinant of change identified was older siblings, that is having one or more older siblings was associated with a greater positive change in TPA and MVPA. The complete set of variables in the final models of PA levels explained a considerable proportion of variance in TPA (28%), MVPA (32%) and SB (20%) and would be associated with a total effect size of about 180 cpm or 30 min/day MVPA. Thirty min of MVPA is half of what children are expected to do at age five or older. Although this is relevant from a guidelines perspective, we are only starting to understand the dose-response relationship of PA with health parameters in preschool children. Nevertheless, in a previous study an increase of 100 cpm was found to be associated with a 2 mmHg lower blood pressure in 5-to-7 year-old children [35]. This reduction was shown to result in a 6% reduction in coronary heart disease risk and a 15% reduction in the risk of stroke and transient ischemic attacks in adults [36]. Furthermore, an increment of 16 min MVPA over 1 year significantly increased fitness, decreased body fat and improved cardiovascular health in schoolchildren [37].

The present study suggests that PA increases in young children. Two previous longitudinal studies also showed an increase in objectively measured physical activity over the preschool years [10, 11]. Jackson DM, Reilly JJ, Kelly LA, Montgomery C, Grant S and Paton JY [10] reported that accelerometer-measured TPA increased by 27% in 60 3-to-4 year-old Scottish children over 1 year. Examining the 24-month follow up in 42 children of the same study, Kelly LA, Reilly JJ, Jackson DM, Montgomery C, Grant S and Paton JY [11] found a 22% increase in activity from baseline. In contrast, a prospective study of 208 preschool aged children in New Zealand found a decline of about 40% in overall and 50% in MVPA in both sexes from age three to age four, without a significant further decline between 4 and 5 years [12]. The variation in results is difficult to explain. Children participating in the aforementioned studies [10–12] were subject to a comparable educational system as children in our study. Since the transition from preschool to primary school is commonly thought to have a marked impact on PA behavior [38], we did not mention studies that included children who had entered formal schooling at follow-up. The contradicting findings, however, may arise from methodological discrepancies in PA data collection and processing such as the use of different devices and intensity thresholds leading to comparability issues, or variation in adjustment for confounders [23]. Our study is the largest prospective study examining longitudinal trajectories of PA and SB and we applied the most rigorous accelerometer validity criteria [39]. In addition, the explanatory power of our models was relatively high, ranging from 26 to 32%. Since wear-time did not differ significantly between the age categories and was shown to be unrelated to activity intensities, the increase in PA found in our study was not attributable to the slightly longer wear-time in older children. Rather, a plausible explanation that has been reinforced by relevant existing literature is that advancing motor proficiency in this age group contributes to an increase in PA levels [40–42].

Our results further illustrate that sex differences in physical activity already occur from a young age. We found boys to consistently engage in more TPA and MVPA than girls at all ages during the preschool years, a pattern commonly described in later childhood and adolescence. While TPA and MVPA increased over time in both sexes, the difference in activity levels between the sexes was lowest for the youngest children and with a difference of 16% (115 cpm) for TPA and of 24% (27 min/day) for MVPA highest in the oldest. There is strong evidence from cross-sectional and longitudinal studies pointing towards a sex difference in activity levels at the preschool age [5, 43–46]. Currently, there is no conclusive explanation as to why girls are less physically active than boys and whether these lower levels are a health concern. In fact, considering that overall females have higher health status than men in spite of being less physically active than their male peers throughout the life span [47], it is questionable whether the “one-size-fits-all” approach is appropriate for meaningful and beneficial PA. Males and females may be predisposed to engage in different levels of intensity and type of activity [48]. It is plausible to assume that biological differences, such as patterns of development and maturation and differences in physiology and body composition, may contribute to sex differences in PA. Thus, before we continue targeting females with the aim of increasing PA to recommended standards, a clear understanding of whether and how PA provides health benefits differently for males and females and how findings may translate to policy and practice has to be established.

We further found a strong and consistent association between the child’s activity temperament and objectively measured TPA, MVPA and SB in both boys and girls. Parents reported their children’s temperamental characteristics using the Emotionality, Activity, and Sociability Temperament Survey (EAS) [49] at baseline and follow-up. Of all four dimensions used to conceptualize temperament (emotionality, activity, and sociability and shyness), only the activity dimension emerged as a predictor of PA and SB. Activity temperament refers to an individual’s preferred level of activity and speed of action. As temperamental traits reflect generalized tendencies rather than motivation or abilities and are believed to change little over time [50, 51], a young child’s temperamental activity may influence lifelong PA with potential implications for disease risk. Examining the activity temperament may reveal important pre-dispositions for PA and help identify high-risk individuals. Moreover, an understanding of the underlying processes and relationships that link temperament with the development of PA patterns may provide important insights for PA promotion. The role of childhood temperament in shaping activity behavior is still largely unexplored. One previous cross-sectional study concluded that none of the six domains of temperament, including activity, assessed by the Child Temperament Questionnaire (CTQ) was associated with objectively measured PA and SB in preschoolers [52] whereas a recent publication found that early childhood temperamental activity level predicted self-reported adolescent PA in males [53].

Consistent and robust evidence about what elements effect positive change in young children’s PA is lacking to date and limited attention has been given to the impact of siblings. However, as children spend a large amount of time in the home environment, it is important to consider sibling influence. Evidence from cross-sectional studies suggests a positive association between the presence of older siblings in the household and children’s MVPA [54]. A recently published systematic review of qualitative literature concluded that siblings were perceived to both facilitate and inhibit young children’s physical activity levels [55]. On the one hand, preschool aged children often want to mimic or play with their older siblings and siblings may take pressure off parents by providing other children with whom the young child can be active [55, 56]. On the other hand, siblings were found to have no influence on or even be a barrier for preschoolers’ PA because having more than one child may pose challenges to facilitating organized PA or finding activities suitable for children at varying developmental stages [55–57]. While most studies considered the influence of siblings in general, a recent qualitative study in preschool children showed that specifically older siblings tended to influence the younger more often and that particularly home-based PA was positively affected (unless older siblings inspired the younger to participate in structured PA) [57]. Among the scarce quantitative evidence investigating determinants of change in young children’s PA [21], the only prospective study examining sibling influence found that sibling co-participation in PA but not sibling PA levels was positively associated with change in MVPA among five- to six-year-olds [58]. Our findings suggest that siblings play an important role in shaping their younger’s activity behavior by inducing a positive change in TPA and MVPA, irrespective of the absolute levels. Interventions aimed at increasing PA levels among young children may have a greater likelihood of success if the potential of sibling influence is considered/included. Increasing parental awareness of free, informal and spontaneous opportunities in home-based settings, e.g. in the garden or neighborhood, may help support the influence of siblings. This is particularly important, as parents of young children are central to facilitating or restricting much of their child’s interaction with the environment, such as the interaction with siblings and peers. Moreover, special emphasis should be placed on firstborns and only-children. In the case where older siblings are not available, interventions may focus on providing opportunities for companionship or playmates to substitute sibling encouragement and role modeling. For instance, children without older siblings from different families may be paired to promote their PA. Further exploration of the unique influence of siblings and friends is required.

Key strengths of the present study include the longitudinal design, which allows for drawing more robust conclusions about the direction of associations and changes over time, the comprehensive assessment of numerous objective measures and parent-report data, the use of objective, reliable, and validated measures of PA, the examination of an extended set of potential determinants guided by the socio-ecological model, and the large and representative sample of preschool children. Our study has several limitations. The relatively short follow-up period of 12 months is one of our key limitations. In addition, accelerometers were removed during aquatic activities likely resulting in an underestimation of PA, and parent-reported data used in this study might have been biased by memory and expectations. Although the proportion of variance explained based on the final models indicate a good performance of the set of exposure variables, particularly for TPA and MVPA, additional factors not considered in this work may have a substantial influence on activity levels in young children. Such factors include, amongst others, genetic traits, variables related to different contexts, policies and practices (e.g. the care environment) and site-specific objectively measured physical environmental properties. Finally, the generalizability of our findings may be reduced because our sample was recruited from children attending childcare on a regular basis and we cannot rule out the possibility that some of the associations observed are chance findings.

## Conclusions

In our sample of typically developing preschool children, PA increased over time with boys being consistently more active than girls. Among a large set of diverse potential determinants, individual-level factors had the greatest influence on activity behavior; age, sex and activity temperament were most relevant to determining activity levels, and the existence of older siblings was found to induce positive change in these levels. Despite enormous efforts, research has failed to identify modifiable determinants of physical activity that can be used to inform physical activity promotion. We therefore suggest that future research should attempt to broaden our understanding of how young children’s sufficient PA levels can be maintained following the preschool years. We urgently need good-quality longitudinal studies that cover the entire range of childhood development including important factors such as educational and migrational transitions, social networks and epigenetics.

## Additional files

Additional file 1: Potential determinants. Detailed description of all potential determinants. (PDF 146 kb)

Additional file 2: Flow chart. Diagram describing the inclusion of participants in the present study. (PDF 54 kb)

## Abbreviations

BMI: Body mass index
cpm: counts per minute
EAS: Emotionality, activity, and sociability temperament survey
ISEI: International socio-economic index
MI: Multiple imputation
min: Minute
MVPA: Moderate-to-vigorous physical activity
OECD: The Organization for Economic Co-operation and Development
PA: Physical activity
SB: Sedentary behavior
SD: Standard deviation
SES: Socio-economic status
TPA: Total physical activity
WHO: World Health Organization
