# Supplementary material for: Physical activity and sedentary behavior in preschoolers: a longitudinal assessment of trajectories and determinants
Source: Int J Behav Nutr Phys Act. 2018 Apr 4;15:35. doi: 10.1186/s12966-018-0670-8 (PMC5885465; doi:10.1186/s12966-018-0670-8)
Supplement: Supplementary file 1 — Potential determinants. Detailed description of all potential determinants. (PDF 146 kb) [file 12966_2018_670_MOESM1_ESM.pdf]

Additional file 1: Potential determinants

Table 1: Detailed description of potential determinants of young children's objectively measured physical activity and sedentary behavior (n=498)

	Variable description and coding	Use in analysis	Mean (SD) or percentages		% misssing	
			T0	T1	T0	T1
**Demographic and biological variables**						
Sex	Child's gender	Binary variable (% boys)	53.8	51.9	None	None
Age	Child's age	Continuous variable (years)	3.9 (0.7)	4.9 (0.7)	None	None
Birth weight	Child's birth weight	Continuous variable (grams)	3290 (570)	3280 (530)	6.0	6.1
Chronic health condition	Whether or not child has a chronic condition	Re-coded to dichotomous variable (% with chronic health condition)	7.5	7.1	4.6	33.5
BMI[a]	BMI-for-age percentiles based on the World Health Organization (WHO) Child Growth Standards [1], categorized as normal (<85th percentile) vs. overweight and obese (≥85th percentile)	Re-coded to dichotomous variable (% overweight or obese)	24.1	18.5	2.4	31.7
Gross motor skills[a]	Composite z-score of four individual sub-scores (walking, running, jumping, and hopping), assessed using the Zurich Neuromotor Assessment [2, 3]	Continuous variable (composite z-score)	0.0 (1.0)	0.1 (1.1)	16.1	34.5
Siblings	Presence of older siblings in household	Binary variable (% having older siblings)	43.2	44.5	3.6	3.8
Parental BMI	Parental BMI divided into both parents normal- vs. at least one parent overweight or obese	Re-coded to dichotomous variable (% at least one overweight or obese parent)	52.2	52.5	8.0	36.5
SES	SES based on mother's or father's occupation (depending on who is highest) using the International Socio-Economic Index (ISEI) coding [4]	Continuous variable (parental ISEI score [range 16–90; increases with higher SES])	63.1 (15.5)	62.7 (15.6)	4.4	16.3
Family structure	Single-parent vs. dual-parent household	Binary variable (% single parent households)	10.4	9.8	5.4	34.1
**Psychological, cognitive and emotional variables**						
Self-regulation[a]	Assessed using the Statue Test of the Neuropsychological Assessment for Children (NEPSY) [5]	Continuous variable (NEPSY score [range 0-30; increases with better self-regulation])	19.2 (9.5)	23.3 (8.0)	14.3	32.7
Psychological difficulties	Assessed using the Strengths and Difficulties Questionnaire (SDQ) [6], calculated as the sum of scores on the emotional, conduct, hyperactivity and peer relationship problem scales	Continuous variable (SDQ total score [range 0-40; increases with more difficulties])	8.8 (4.3)	8.5 (4.7)	6.6	35.7
Emotionality temperament	Assessed using the Emotionality, Activity, and Sociability Temperament Survey (EAS) [7, 8], calculated as the average of five items capturing the tendency to become aroused easily and intensely	Continuous variable (EAS emotionality score [range 1-5; increases with more pronounced trait])	2.8 (0.7)	2.8 (0.7)	6.6	36.1

Activity temperament	Assessed using the Emotionality, Activity, and Sociability Temperament Survey (EAS) [7, 8], calculated as the average of five items capturing the preferred levels of activity and speed of action	Continuous variable (EAS activity score [range 1-5; increases with more pronounced trait])	3.8 (0.7)	3.7 (0.7)	6.6	36.1
Shyness temperament	Assessed using the Emotionality, Activity, and Sociability Temperament Survey (EAS) [7, 8], calculated as the average of five items capturing the tendency to be inhibited and awkward in new social situations	Continuous variable (EAS shyness score [range 1-5; increases with more pronounced trait])	2.4 (0.7)	2.4 (0.7)	6.6	36.1
Parenting stress	Assessed using the Parenting Stress Scale (PSS) [9], calculated as the sum of scores on the 18-item questionnaire	Continuous variable (PSS score [range 5-90; increases with more parenting stress])	37.5 (7.5)	37.6 (7.6)	6.6	36.3
Cognitive performance[a]	Composite z-score of four individual sub-scores (perception, attention, memory, and reasoning) assessed using the Intelligence and Development Scales – Preschool (IDS-P) [10]	Continuous variable (composite z-score)	0.0 (1.0)	0.1 (0.9)	5.6	31.9
**Behavioral variables**						
Sleep duration	Hours of sleep/night averaged over a week	Continuous variable (hours)	10.8 (0.6)	10.8 (0.5)	4.6	33.7
Play frequency	Child plays more than once per week vs. equal to or less than once per week with other children (sibling, friend, neighbor)	Re-coded to dichotomous variable (% more than once/week)	91.2	93.1	5.0	34.1
**Social and cultural variables**						
Parental sedentary behavior	Average hours/day parents spends on screen-based or other sedentary activity	Continuous variable (hours)	2.8 [2.0, 4.5][b]	3.0 [2.0, 5.0][b]	5.2	33.9
Parental sports club membership	At least one parent vs. none of parents has a sports club membership	Re-coded to dichotomous variable (% at least one parent is member)	32.0	32.8	7.2	34.4
Parental physical activity	At least one parent vs. none of parents engages in ≥150min MVPA per week [11]	Re-coded to dichotomous variable (% at least one parent is active)	63.4	61.6	4.6	33.9
Parental Involvement in child physical activity	At least one parent participates in ≥1 organized PA or ≥2 non-organized PAs with child per week vs. none of parents participates in the activities described above	Re-coded to dichotomous variable (% at least one parent is involved)	65.0	52.3	9.4	34.1
Mode of transport to childcare	Active transport (walking) vs. passive transport to childcare	Binary variable (% active)	40.7	48.1	5.4	33.7
Parental tobacco use	At least one parent vs. none of parents smokes	Re-coded to dichotomous variable (% at least one parent smokes)	24.1	24.4	5.6	33.7
Parental alcohol consumption	Presence of a parent consuming alcohol in amounts greater than the recommended intake levels (males: 2 drinks/day, females: 1 drink/day [12, 13])	Re-coded to dichotomous variable (% at least one parent consumes large amounts)	5.5	4.5	4.8	33.3
**Environmental variables**						
Time outdoors	Average hours per day child spends outdoors	Continuous variable (hours)	2.0 [1.5, 3.0][b]	2.0 [1.5, 3.0][b]	7.2	38.6

Fixed toys	Number of fixed play items at home (in-and outside)	Continuous variable (number of items [range 0-7])	1.6 (1.5)	1.6 (1.6)	3.2	32.9
Portable toys	Number of portable play items at home (in-and outside)	Continuous variable (number of items [range 0-8])	4.5 (1.5)	4.3 (1.5)	3.2	32.9
Days at childcare	Number of days per week child is in childcare	Continuous variable (number of days [range 0-5])	2.8 (1.2)	2.5 (1.3)	3.8	37.6
Living area per person	Size of total indoor space per person	Continuous variable (m^2)	30.8 (9.1)	30.8 (8.9)	6.8	34.1
Neighborhood safety	Parental perception of neighborhood safety measured using an 11-item questionnaire (based on [14-16]) on traffic density, road safety, crime, strangers and access to outdoor play facilities in the local area	Continuous variable (neighborhood safety sum score [range 0-44; increases with increasing concerns])	12.6 (7.0)	12.4 (7.2)	9.4	38.8
Dog	Whether or not there is a dog in the household	Binary variable (% dog owner)	6.9	5.5	6.0	33.3
Season	Data collection conducted in summer vs. spring and autumn	Re-coded to dichotomous variable (% spring and fall months)	75.3	77.9	None	27.9
Region	Urban vs. rural (urban defined as ≥50000 inhabitants [17])	Binary variable (% urban)	34.9	39.4	None	27.9

[a]Directly measured (all other information is parent-report)

[b]Median and inter-quartile range presented for skewed distribution

PA, physical activity; SB, sedentary behavior; BMI, Body mass index; SES, socio-economic status

References

1. WHO Multicentre Growth Reference Study Group: **WHO Child Growth Standards: Length/height-for-age, weight-for-age, weight-for-length, weight-for-height and body mass index-for-age: Methods and development.** Geneva: World Health Organization; 2006.
2. Kakebeeke TH, Caflisch J, Chaouch A, Rousson V, Largo RH, Jenni OG: **Neuromotor development in children. Part 3: motor performance in 3- to 5-year-olds.** *Dev Med Child Neurol* 2013, **55:**248-256.
3. Kakebeeke TH, Locatelli I, Rousson V, Caflisch J, Jenni OG: **Improvement in gross motor performance between 3 and 5 years of age.** *Percept Mot Skills* 2012, **114:**795-806.
4. Ganzeboom HBG, De Graaf PM, Treiman DJ: **A standard international socio-economic index of occupational status.** *Social Science Research* 1992, **21:**1-56.
5. Korkman M, Kirk U, Kemp S: *NEPSY: A developmental neuropsychological assessment.* Psychological Corporation; 1998.
6. **Strengths and Difficulties Questionnaire (SDQ): information for researchers and professionals about the Strengths and Difficulties Questionnaire** [http://www.sdqinfo.com/]
7. Buss AH, Plomin R: *Temperament: Early developing personality traits.* Lawrence Erlbaum; 1984.
8. Spinath FM: **Temperamentsmerkmale bei Kindern.** *Zeitschrift für Differentielle und Diagnostische Psychologie* 2000, **21:**65-75.
9. Berry JO, Jones WH: **The Parental Stress Scale - Initial Psychometric Evidence.** *Journal of Social and Personal Relationships* 1995, **12:**463-472.
10. Grob A, Meyer CS, Hagmann-von Arx P: *Intelligence and Development Scales (IDS).* Bern: Hans Huber; 2009.
11. World Health Organization: **Global recommendations on physical activity for health.** Geneva: World Health Organization, ; 2010.
12. Schweizerische Gesellschaft für Ernährung: **Merkblatt Alkohol.** Berne, Switzerland; 2011.
13. U.S. Department of Health and Human Services and U.S. Department of Agriculture: **2015 – 2020 Dietary Guidelines for Americans.** 8th edition; 2015.
14. Saelens BE, Sallis JF, Black JB, Chen D: **Neighborhood-based differences in physical activity: An environment scale evaluation.** *American Journal of Public Health* 2003, **93:**1552-1558.
15. Timperio A, Crawford D, Telford A, Salmon J: **Perceptions about the local neighborhood and walking and cycling among children.** *Preventive Medicine* 2004, **38:**39-47.
16. Weir LA, Etelson D, Brand DA: **Parents' perceptions of neighborhood safety and children's physical activity.** *Preventive Medicine* 2006, **43:**212-217.
17. OECD: **Redefining "Urban": A New Way to Measure Metropolitan Areas.** OECD Publishing; 2012.
