# Supplementary figures and images for: Physical activity and sedentary behavior in preschoolers: a longitudinal assessment of trajectories and determinants
Source: Int J Behav Nutr Phys Act. 2018 Apr 4;15:35. doi: 10.1186/s12966-018-0670-8 (PMC5885465; doi:10.1186/s12966-018-0670-8)

## Additional file 2. Flow Chart

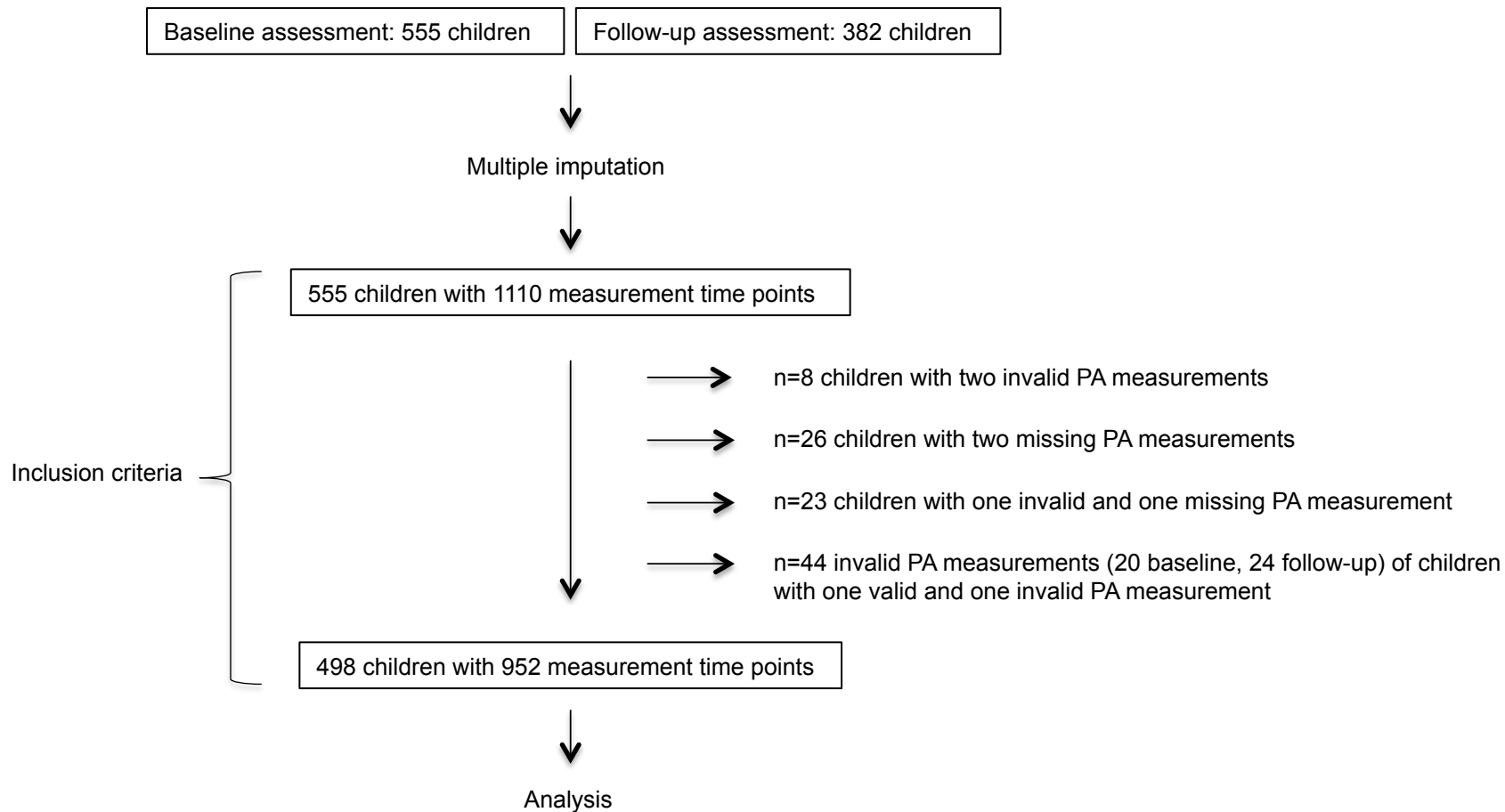

Supplement: Supplementary file 2 — Flow chart. Diagram describing the inclusion of participants in the present study. (PDF 54 kb) [file 12966_2018_670_MOESM2_ESM.pdf]
